# Horizontal Gene Transfer of Fluoroquinolone Resistance-Conferring Genes From Commensal *Neisseria* to *Neisseria gonorrhoeae*: A Global Phylogenetic Analysis of 20,047 Isolates

**DOI:** 10.3389/fmicb.2022.793612

**Published:** 2022-03-17

**Authors:** Sheeba Santhini Manoharan-Basil, Natalia González, Jolein Gyonne Elise Laumen, Chris Kenyon

**Affiliations:** ^1^Department of Clinical Sciences, Institute of Tropical Medicine Antwerp, Antwerp, Belgium; ^2^Department of Medicine, University of Cape Town, Cape Town, South Africa

**Keywords:** HGT, *gyrA*, *gyrB*, *parC*, *parE*, *Neisseria gonorrhoeae*, commensal *Neisseria*

## Abstract

Antimicrobial resistance in *Neisseria gonorrhoeae* is an important global health concern. The genetically related commensal *Neisseria* act as a reservoir of resistance genes, and horizontal gene transfer (HGT) has been shown to play an important role in the genesis of resistance to cephalosporins and macrolides in *N. gonorrhoeae*. In this study, we evaluated if there was evidence of HGT in the genes *gyrA/gyrB* and *parC/parE* responsible for fluoroquinolone resistance. Even though the role of *gyrB* and *parE* in quinolone resistance is unclear, the subunits *gyrB* and *parE* were included as zoliflodacin, a promising new drug to treat *N. gonorrhoeae* targets the *gyrB* subunit. We analyzed a collection of 20,047 isolates; 18,800 *N. gonorrhoeae*, 1,238 commensal *Neisseria* spp., and nine *Neisseria meningitidis*. Comparative genomic analyses identified HGT events in genes, *gyrA*, *gyrB*, *parC*, and *parE*. Recombination events were predicted in *N. gonorrhoeae* and *Neisseria* commensals. *Neisseria lactamica*, *Neisseria macacae*, and *Neisseria mucosa* were identified as likely progenitors of the HGT events in *gyrA*, *gyrB*, and *parE*, respectively.

## Introduction

*Neisseria gonorrhoeae*, a gram-negative diplococcus, is an obligate human pathogen that causes the sexually transmitted infection gonorrhea (Ligon, 2005; Tapsall et al., 2009). *Neisseria gonorrhoeae* has been remarkably adept at developing resistance to antimicrobials, including β-lactams, macrolides, and fluoroquinolones. This has resulted in *N. gonorrhoeae* being included as a high priority pathogen by the WHO (Willcox, 1970; CDC, 1985; Unemo and Shafer, 2011; Chesson et al., 2014; Blair et al., 2015; Tacconelli and Magrini, 2017). Current treatment guidelines typically advocate using only ceftriaxone or in combination with azithromycin (Unemo et al., 2020). Increasing incidence of resistance to both ceftriaxone and azithromycin have led to novel treatment strategies. A particularly promising novel agent, zoliflodacin, a spiropyrimidinetrione, whose target site is *gyrB* is currently being evaluated in phase III studies following successful phase II studies (Alm et al., 2015; Taylor et al., 2018; Bradford et al., 2020). Recent trials have also established that ciprofloxacin (CIP) can be used to treat gonorrhea if genotyping confirms the absence of *gyrA* mutations (Allan-Blitz et al., 2017). This renewed interest in agents targeting the DNA gyrases led to the current study to assess if *gyrA/B* and *parC/E* mutations in *N. gonorrhoeae* can be acquired by horizontal gene transfer (HGT).

The first-generation quinolone, nalidixic acid, was first used in 1962 (Lesher et al., 1962). The second-generation fluoroquinolone, CIP, was first used clinically in the mid-1980s, and from 1993, it was used to treat uncomplicated gonorrhea (CDC, 1993; Davis et al., 1996). Fluoroquinolones target DNA gyrase (topoisomerase II) and topoisomerase IV, the enzymes involved in the DNA replication process (Drlica and Zhao, 1997; Larkin et al., 2007). During DNA replication, DNA gyrase catalyzes the unwinding of DNA molecules and topoisomerase IV decatenates daughter chromosomes following DNA replication (Reece and Maxwell, 1991; Kato et al., 1992; Zechiedrich and Cozzarelli, 1995; Zechiedrich et al., 1997). DNA gyrase is comprised of two subunits, GyrA and GyrB (molecular mass ≈96 and 88 kDa, respectively), which are homologous to the C and E subunits of topoisomerase IV, designated as ParC (molecular mass ≈88 kDa), and ParE (molecular mass ≈70 kDa), respectively. Ciprofloxacin resistance in *N. gonorrhoeae* is caused by point mutations in the quinolone resistance-determining region (QRDR) of *gyrA* and *parC*, which results in amino acid substitutions that alter the target protein structure, thereby reducing the fluoroquinolone-target enzyme binding affinity, leading to resistance (Belland et al., 1994; Piddock, 1999; Alcalá et al., 2003; Bodoev and Il’ina, 2015; Yahara et al., 2020). The majority of the resistance mutations in gonococci are located in the QRDR regions, in codons 67–106 in GyrA and 56–108 in ParC (Belland et al., 1994). The known resistant-associated mutations (RAMs) include substitutions at amino acid positions S91 and D95 in GyrA and G85, D86, S87, S88, Q91, and R116 in ParC (Piddock, 1999; Yang et al., 2006; Bodoev and Il’ina, 2015). Studies have shown that GyrA is the primary target and that ParC is the secondary target for fluoroquinolones in *N. gonorrhoeae* (Wittmann et al., 1973; Belland et al., 1994; Trees et al., 1999; Tanaka et al., 2000; Shultz et al., 2001; Lindbäck et al., 2002).

The genus *Neisseria* includes several commensals and two pathogens—*N. gonorrhoeae* and *Neisseria meningitidis*. *Neisseria* species are naturally competent and transfer DNA to one another (Catlin, 1960; Higashi et al., 2011). HGT of *penA*, *mtrCDE*, *rpsE*, and *rplD* from commensal *Neisseria* has been shown to be important in the genesis of resistance to β-lactams and macrolides in the pathogenic *Neisseria* (Bowler et al., 1994; Shafer, 2018; Wadsworth et al., 2018; Yahara et al., 2018; Fiore et al., 2020; Manoharan-Basil et al., 2021). A study from Shanghai has similarly demonstrated that HGT from *gyrA* in commensal *Neisseria* played a critical role in the genesis of fluoroquinolone resistance in *N. meningitidis* (Chen et al., 2020). HGT from commensal streptococci has also been shown to have played a role in the genesis of fluoroquinolone resistance in *Streptococcus pneumoniae* (Irene et al., 1998; Janoir et al., 1999; José et al., 2000). Recently, Yahara et al., 2021 suggested that fluoroquinolone resistance substitutions at GyrA and ParC in *N. gonorrhoeae* isolates have arisen from independent mutations and evidence of HGT in *gyrA/B* and *parC/E* were not available for *N. gonorrhoeae*. It is unknown but essential to establish if HGT has played a role in fluoroquinolone resistance in *N. gonorrhoeae*. If commensal *Neisseria* can serve as a reservoir of resistance for *N. gonorrhoeae*, then addressing the resistance determinants in commensals is important (de Korne-Elenbaas et al., 2021; Kenyon et al., 2021). The above considerations led to the current study, where we used a dataset of 20,047 globally sourced *N. gonorrhoeae* (1928–2020), *N. meningitidis* and *Neisseria* commensals to assess if there was evidence of HGT in the four fluoroquinolone resistance-conferring genes (*gyrA*/*B* and *parC/E*). This led to the identification of HGT in the QRDR regions of *gyrA*, *gyrB*, and *parE* in *N. gonorrhoeae*.

## Materials and Methods

### Dataset for Analysis

The isolates for the analyses were downloaded from the Pathosystems Resource Integration Center V3.6.9 (PATRIC) database (Davis et al., 2020), GenBank,^1^ pubMLST (Jolley et al., 2018), and pathogenwatch.^2^ Whole-genome sequence (WGS) data from our previous study with an additional 8,388 isolates were used in the current study (Manoharan-Basil et al., 2021). WGS data comprised 20,047 isolates, of which 18,800 were *N. gonorrhoeae*, nine were *N. meningitidis*, and 1,238 were commensal *Neisseria* isolates. Supplementary Table 1 in Data Sheet 1 summarizes the organisms used in this study. The European Committee on Antimicrobial Susceptibility Testing breakpoints was used to define ciprofloxacin susceptibility.^3^ The minimum inhibitory concentration (MIC) to ciprofloxacin (CIP) ≥16 mg/L, >0.06 mg/L, 0.03 ≤ 0.06, and <0.03 mg/L are classified as being high-level resistant (HLR), resistant (R), intermediate (I), and susceptible (S) to CIP, respectively (Sánchez-Busó et al., 2021). The isolates were categorized into three distinct eras: pre-antibiotic (pre-1950s), golden (1950–1970s), and post-modern (1980-20-first century) following the convention of Golparian et al. (2020).

### Gene-By-Gene Analysis, Allelic Profiling, and Recombination Analysis

The analysis was carried out as described in Manoharan-Basil et al. (2021). In brief, WGS (*n* = 20,047) were analyzed using chewBBACA version 2.8.5 (Silva et al., 2018), followed by recombination analysis using Recombination Detection Program (RDP4) program version 4.100 (Martin et al., 2015). Firstly, a training file was created from the complete genome of *N. gonorrhoeae* FA1090 using Prodigal and was used in subsequent steps (Hyatt et al., 2010). Secondly, a study-specific *Neisseria* scheme was created from two complete *Neisseria gonorrhoeae* genomes (FA1090 and MS11). Thirdly, a FASTA file for each coding sequence (CDS) was generated, followed by the creation of whole-genome (wg) multi-locus sequence typing (MLST) loci. The core-genome (cg) MLST loci were then extracted from the wgMLST loci and visualized using a grape tree (Zhou et al., 2018). GrapeTree facilitates the clustering of the isolates based on their allelic profiles using a minimum spanning algorithm. The SchemaEvaluator option implemented in chewBBACCA uses MAFFT that allows multiple sequence alignments of the alleles of each locus and constructs neighbor-joining (NJ) tree using ClustalW2 (Katoh et al., 2002; Larkin et al., 2007). Finally, UniProtFinder^4^ was used to retrieve the functional information of the CDSs. *GyrA*, *gyrB*, *parC*, and *parE* genes were identified based on the UniProt identifier. The multiple sequence alignments and NJ trees of the above genes were extracted from the schema evaluator. The gene-by-gene analysis was carried out on a server with Intel(R) Xeon(R) Silver 4114 CPU @ 2.20 GHz with 125 Gb RAM and HDD distributed RAID-5 using 20 CPU. The analysis took ~15 days to complete.

The multiple sequence alignment files were imported in MEGAX version 10.1.7, and the CDSs were translated (Kumar et al., 2018). The presence or absence of non-synonymous substitutions in the QRDR regions for each amino acid position was denoted as “0” and “1,” respectively. The NJ trees and the corresponding metadata were visualized using Interactive tree of life (iTOL) v6 (Letunic and Bork, 2021) and microreact (Argimón et al., 2016).

Whole-genome sequence of commensal *Neisseria* spp., (*n* = 1,247) along with *N. meningitidis* (*n* = 9) and *N. gonorrhoeae* (*n* = 18,800) were used in the recombination analysis (Supplementary Table 1 in Data Sheet 1). The nucleotide alignments of each gene were screened for recombination events using the Recombination Detection Program (RDP4) program (Martin et al., 2015). Recombinant events supported by at least two of the seven algorithms available in RDP software: RDP, GENECONV, Bootscan, Maxchi, Chimera, SiSscan, and 3Seq were used with default settings, except the window size was increased to 60 nt in RDP, 120 nt in MaxChi and Chimera, and to 500 in BootScan and SiScan (Lemey et al., 2009; Gomez-Valero et al., 2011; Manoharan-Basil et al., 2021). NJ trees were constructed using 1,000 bootstrap replicates (Salminen et al., 1995). According to the definition in RDP4, the minor parent is the parental sequence that contributes the smaller fraction of the recombinant sequence, while the major parent is the parental sequence that contributes the larger fraction (Martin et al., 2015). Additionally, for the sequences which had the same recombination event, the percentage similarities of the nucleotide were determined between donor and recipient sequences. Furthermore, to identify the RAM harboring donors of the *gyrA* and *parC* in *N. gonorrhoeae* and *Neisseria* commensal isolates, RDP4 analysis was carried out as above and was limited to all *Neisseria* commensal and *N. gonorrhoeae* alleles with known RAMs.

### Statistical Analysis

Statistical analyses were performed using JMP®, version 14.0.0 (SAS Institute Inc., Cary, NC, 1989–2021). To evaluate the correlation between multiple pairs of variables (SNPs) and to assess the correlations between phenotypic and genotypic patterns of resistance to ciprofloxacin, Spearman’s rank correlation was used (Elhadidy et al., 2020). Geometric mean (GM) MICs were calculated for each allele. A value of *p* of <0.001 was considered statistically significant.

## Results

### Characteristics of Isolates Used in the Study

**Figure 1:**
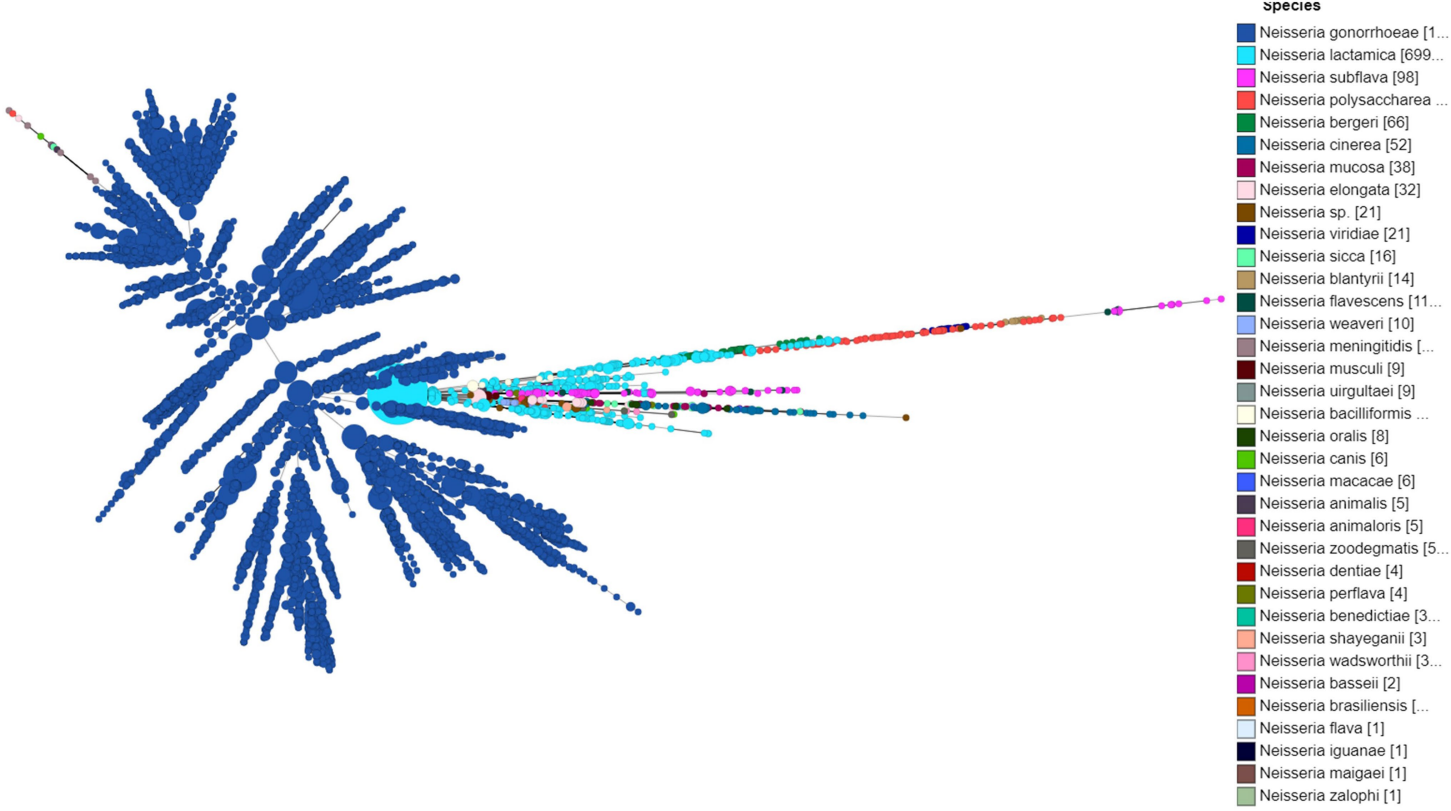
Minimum spanning tree comparing core-genome allelic profiles in association with the *Neisseria* species used in the study. Numbers in brackets refer to the number of isolates. Isolates are displayed as circles. The size of each circle indicates the number of isolates of this particular type.

A total of 20,047 isolates from 1928 to 2020 were used in the study and included 18,800 (93.7%) *N. gonorrhoeae*, nine (0.47%) *N. meningitidis*, and 1,247 (6.2%) *Neisseria* commensal isolates. *Neisseria* commensal included the human nasopharyngeal commensals *N. polysaccharea* (*n* = 74), *N. bergeri* (*n* = 66), *N. lactamica* (*n* = 699), *N. cinerea* (*n* = 52), *N. subflava* (*n* = 98), *N. oralis* (*n* = 8), *N. mucosa* (*n* = 38), *N. elongata* (*n* = 32), and *N. bacilliformis* (*n* = 8), along with several species isolated from animals and animal-related source (*n* = 163; Supplementary Table 1 in Data Sheet 1; Figure 1).

**Figure 2:**
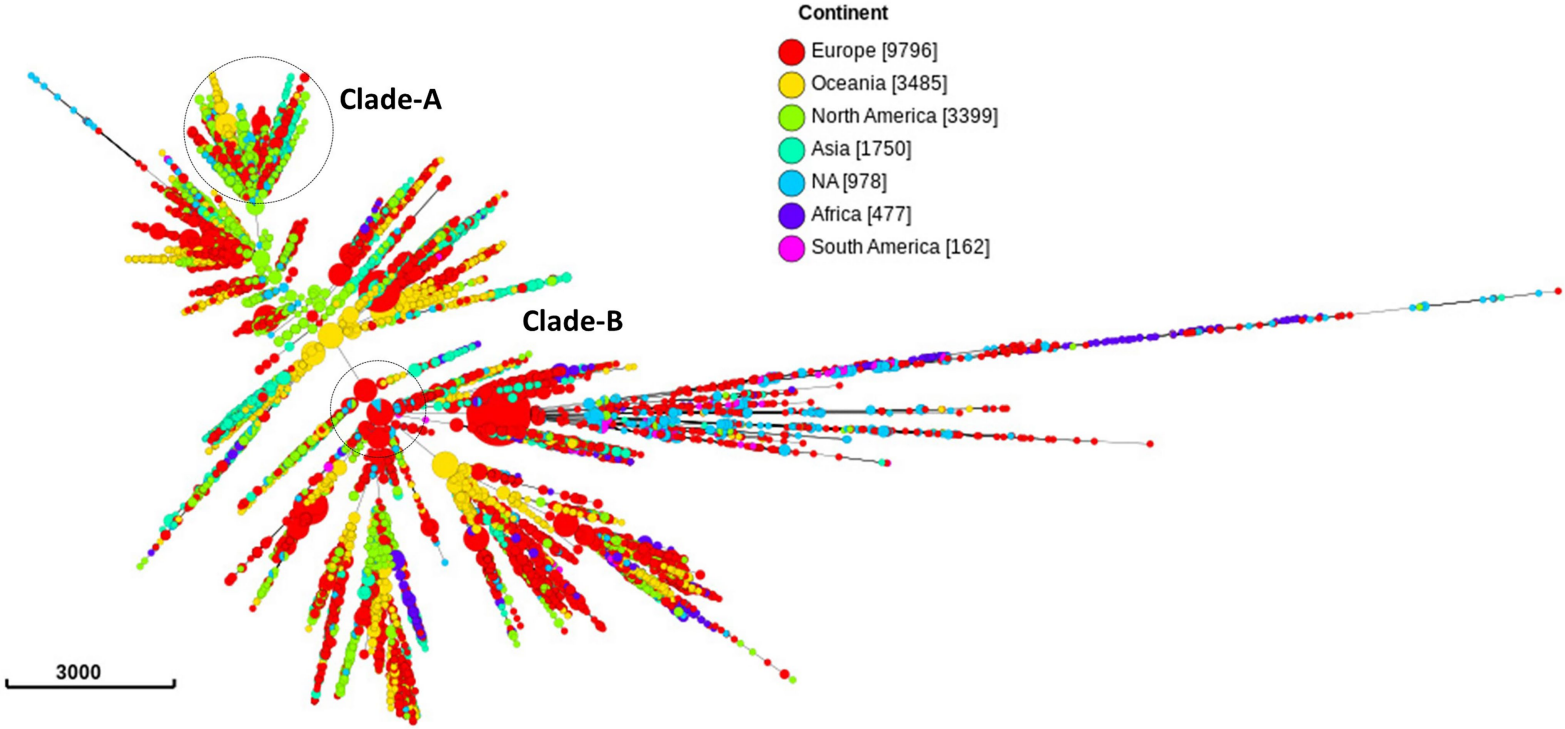
Minimum spanning tree comparing core-genome allelic profiles in association with continents. Numbers in brackets refer to the number of isolates. Isolates are displayed as circles. The size of each circle indicates the number of isolates of this particular type. Clade A, B of ST1901 are represented as dotted circles.

Out of the 20,047 isolates, 9,796 (49.8%) samples were from 31 European countries, 3,399 (16.9%) from North America, 3,485 (17.3%) from Oceania, 1,750 from Asia (8.7%), 477 (2.37%) from Africa, and 162 (0.8%) from South America (Figure 2; Supplementary Table 2 in Data Sheet 1). The country of isolation was not available for 978 (4.8%) isolates.

### Antimicrobial Susceptibility of *Neisseria* Isolates

Among the 18,800 *N. gonorrhoeae* isolates, the CIP MIC was available for 12,942 (68.8%) isolates. Out of these isolates, 6,192 (47.8%) of the isolates were CIP resistant (R), 45 (0.34%) isolates were CIP intermediate (I), and 6,705 (51.8%) isolates were susceptible to CIP (Supplementary Table 2 in Table 2). Out of the 1,247 *Neisseria* commensal species, CIP MIC was available for only 14 isolates. Seven of these isolates had MICs >0.06 mg/L.

### Characterization of Resistance-Associated Mutations in QRDR Regions of *gyrA*, *gyrB*, *parC*, and *parE* Genes in *Neisseria gonorrhoeae*

Strong positive correlations between CIP MICs and substitutions in GyrA at amino acid positions 91 (S91F, 45.4%) and 95 (D95A, 13.82%; D95G, 28.2%; D95N, 3.04%) and ParC at 85 (G85C, 0.5%; G85D, 0.2%), 86 (D86N, 6.8%), 87 (S87I, 1%; S87N, 3.4%; S87R, 25.7%), 88 (S88P, 2.1%), and 91 (E91G, 6.3%; E91K, 0.7%; E91Q, 0.9%) were observed (Supplementary Table 3 in Data Sheet 1).

*In vitro* generated zoliflodacin-resistant mutants containing substitutions in GyrB (D429, K450, and S467N) have been found to be associated with increased MICs of zoliflodacin (Alm et al., 2015; Foerster et al., 2015, 2019). In the current study, substitutions in GyrB-D429V were present in a single Japanese isolate (PubMLST ID-31741; Pathogenwatch ID-ERR363582; Adamson et al., 2021) along with GyrA-S91F and ParC-S87R substitutions. Fifty isolates had the GyrB-S467N substitution [allele 293 (*n* = 12) and allele-446 (*n* = 38); Pathogenwatch (*n* = 24) and PubMLST (*n* = 26)] along with mutations in *gyrA* and or *parC*. These isolates were from China (*n* = 1, ST7367), Japan (*n* = 2, ST7363), Norway [*n* = 11; ST1925 (*n* = 10), ST7363 (*n* = 1)], and Vietnam [*n* = 36; ST7373 (*n* = 34), ST1925 (*n* = 2)]. None of the isolates had the GyrB-K450 substitution.

**Figure 3:**
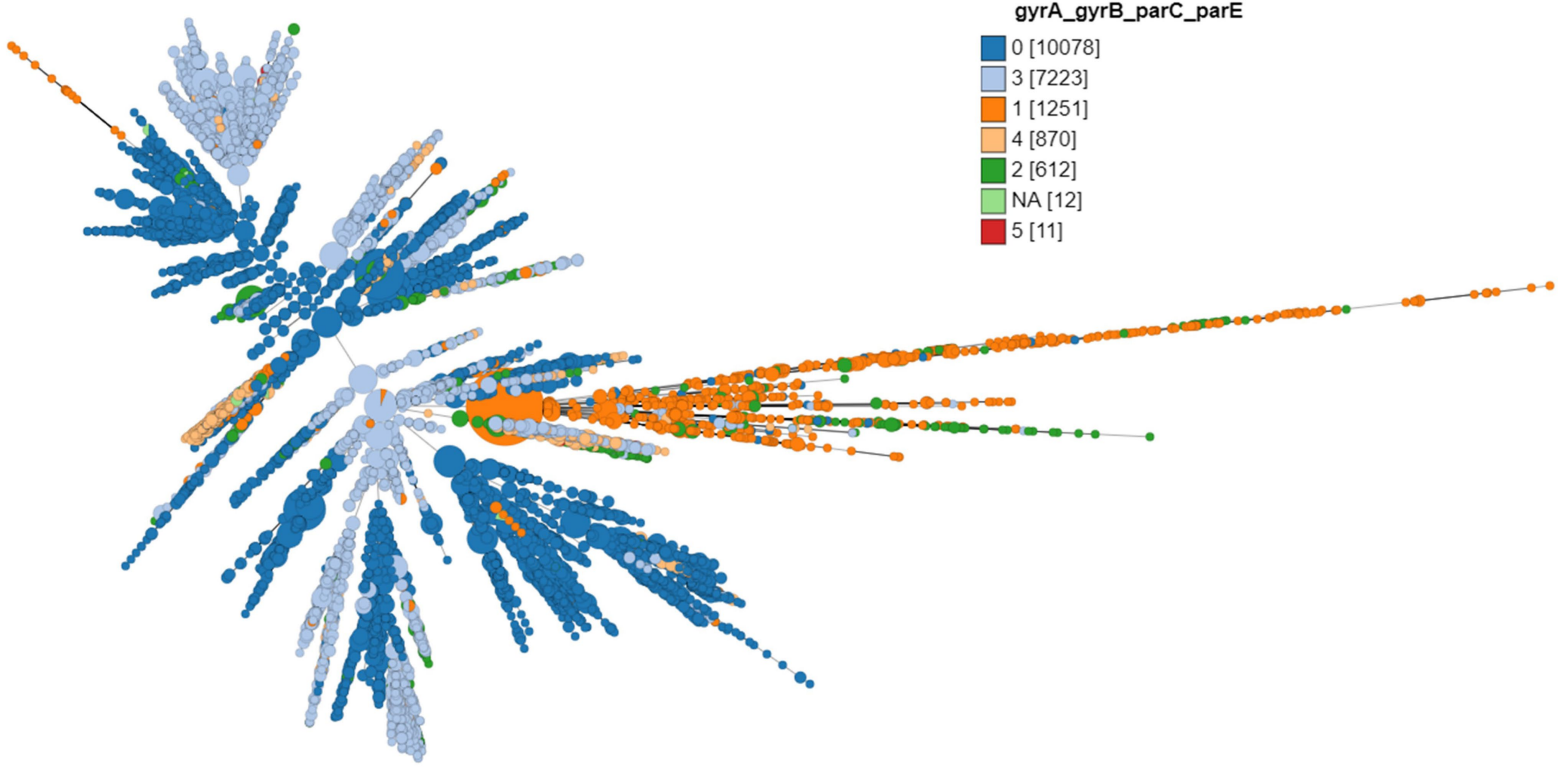
Minimum spanning tree comparing core-genome allelic profiles with the number of substitutions in GyrA, GyrB, ParC, and ParE. Numbers in brackets refer to the number of isolates. Isolates are displayed as circles. The size of each circle indicates the number of isolates of this particular type.

**Figure 4:**
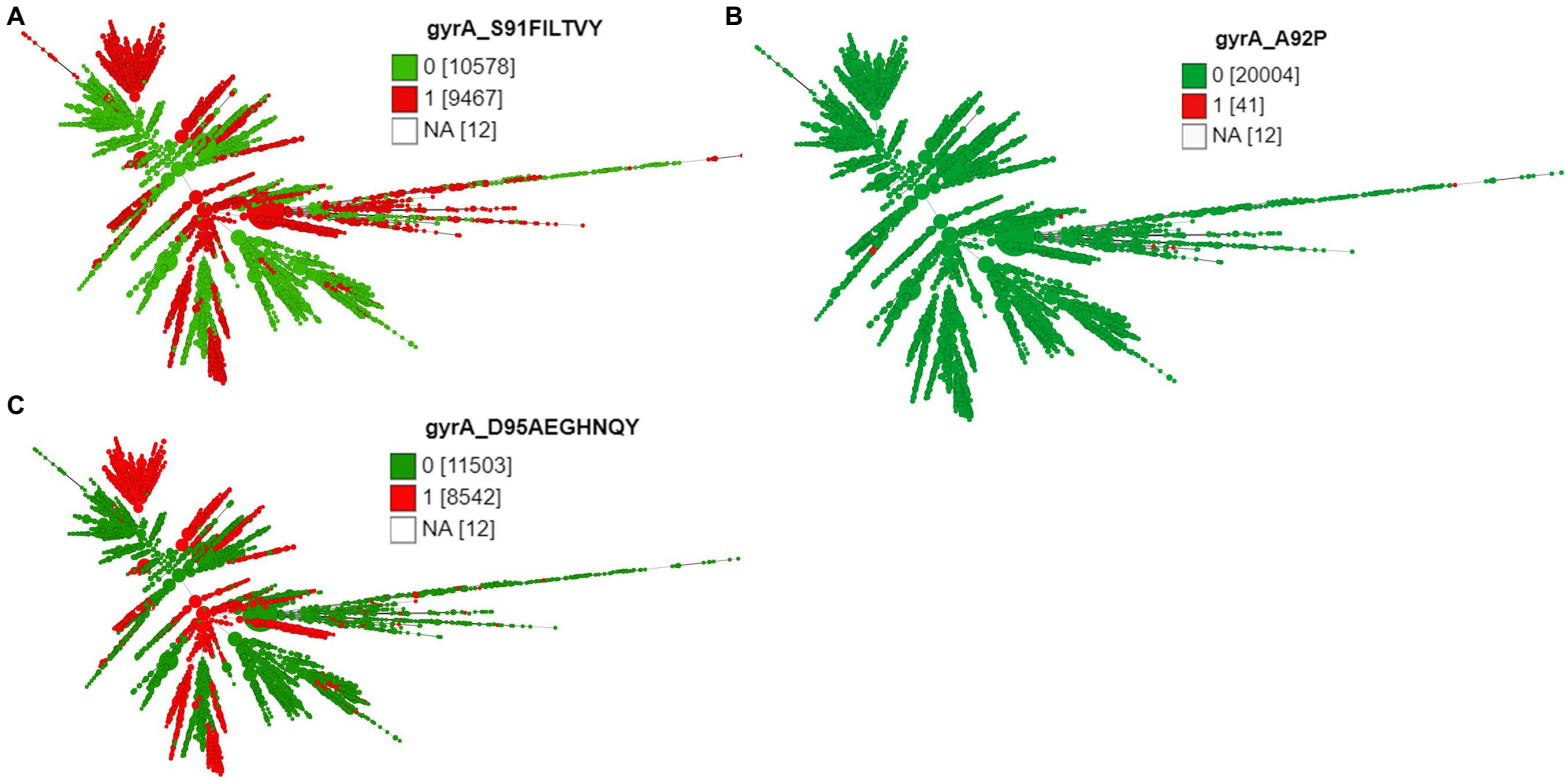
Minimum spanning tree comparing core-genome allelic profiles with GyrA substitutions at amino acid **(A)** position 91, **(B)** position 92, and **(C)** position 95. Numbers in brackets refer to the number of isolates. 1 and 0 denotes the presence and absence of RAMs, respectively. Isolates are displayed as circles. The size of each circle indicates the number of isolates of this particular type.

**Figure 5:**
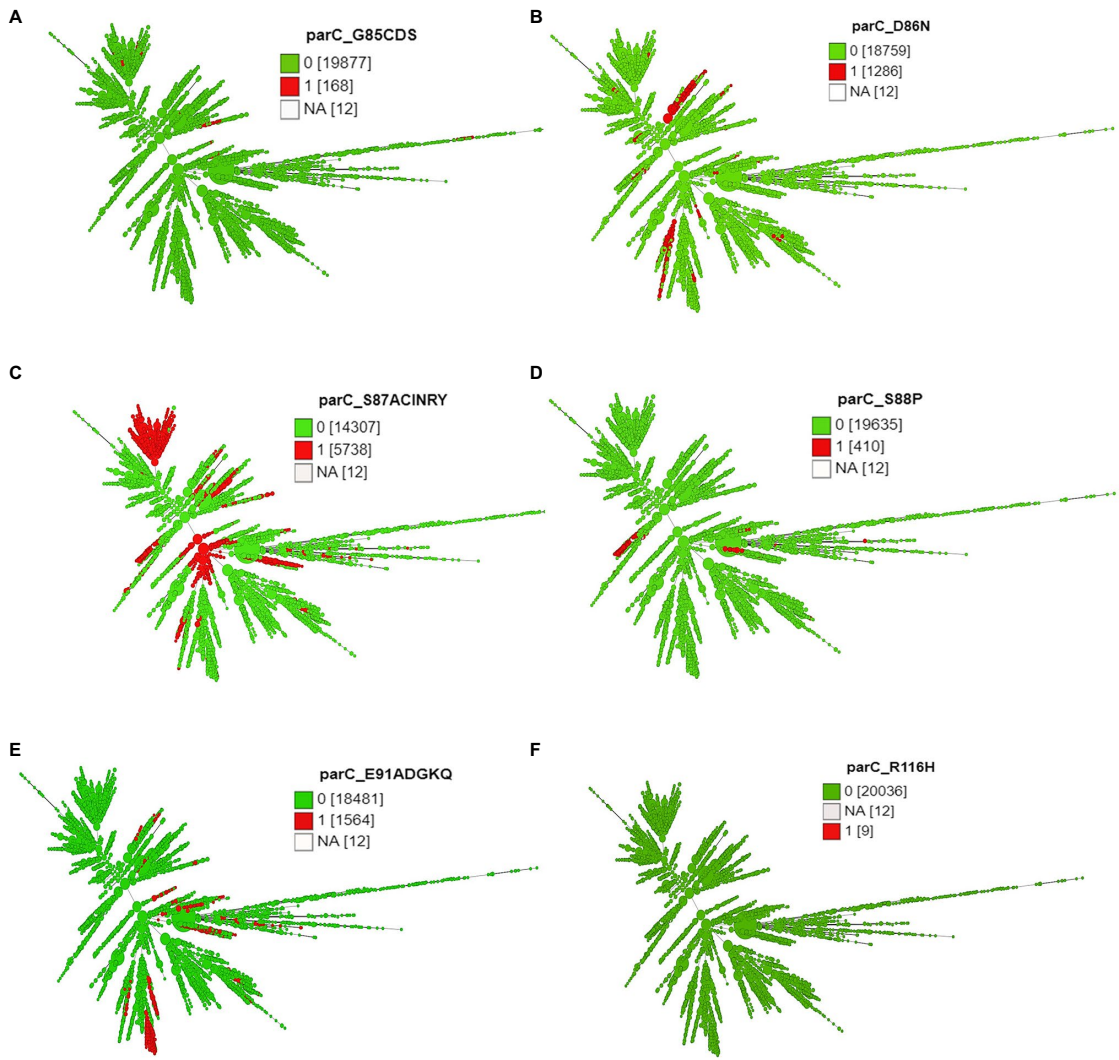
Minimum spanning tree comparing core-genome allelic profiles with ParC substitutions at amino acid **(A)** position 85, **(B)** position 86, **(C)** position 87, **(D)** position 88, **(E)** position 91, and **(F)** position 116. Numbers in brackets refer to the number of isolates. 1 and 0 denotes the presence and absence of RAMs, respectively. Isolates are displayed as circles. The size of each circle indicates the number of isolates of this particular type.

Except 27 isolates, all high-level fluoroquinolone (CIP MICs ≥16 mg/L) resistant gonococcal isolates, *n* = 2,384 (12.7%) had known substitutions at GyrA-91 (S91F), GyrA-92 (A92P), GyrA-95 (D95A/G/N), and ParC-85 (G85C), 86 (D86N), 87 (S87I/N/R/Y), 88 (S88P), and 91 (E91G/K) positions (Figures 3–5; Supplementary Table 2 in Table 2). Twenty-seven isolates (1.1%; GM MIC = 22.3 mg/L), 14 (0.5%; GM MIC = 27.5 mg/L), 2,040 (85%; GM MIC = 21.1 mg/L), 273 (11.4%), and 3 (0.1%) had one, two, three, four, and five RAMs, respectively (Supplementary Table 1 in Table 1). A total of 3,808 (20.2%) *N. gonorrhoeae* isolates had low level fluoroquinolone resistance (CIP MICs >0.06 and <16 mg/L). Out of these isolates, 79 isolates (2%, GM MIC = 1.53 mg/L) had no known RAMs (Figures 3–5; Supplementary Table 2 in Table 2). Eighty-nine (2.3%; GM MIC = 0.64 mg/L), 302 (7.9%; GM MIC = 27.5 mg/L), 3,017 (79.2%; GM MIC = 21.18 mg/L), 326 (8.5%), and 5 (0.1%) isolates had one, two, three, four, and five RAMs, respectively (Supplementary Table 1 in Table 1). Out of 6,705 (35.7%) susceptible gonococcal isolates, 6,620 (98.7%) had no GyrA or ParC substitutions, whereas 46, 9, 33, and 2 isolates had one, two, three, and four RAMs, respectively (Supplementary Table 1 in Table 1). Seven *Neisseria* commensals (0.08%) with ciprofloxacin MICs >0.06 mg/L, had GyrA-S91I/T/V or GyrA-D95N substitutions.

The different combinations of RAMs and the number of isolates are presented in Figures 3–5 and Supplementary Figure 1.

### Genetic Structure and Geographical Dispersal of Fluoroquinolone Resistance in *Neisseria gonorrhoeae*

**Figure 6:**
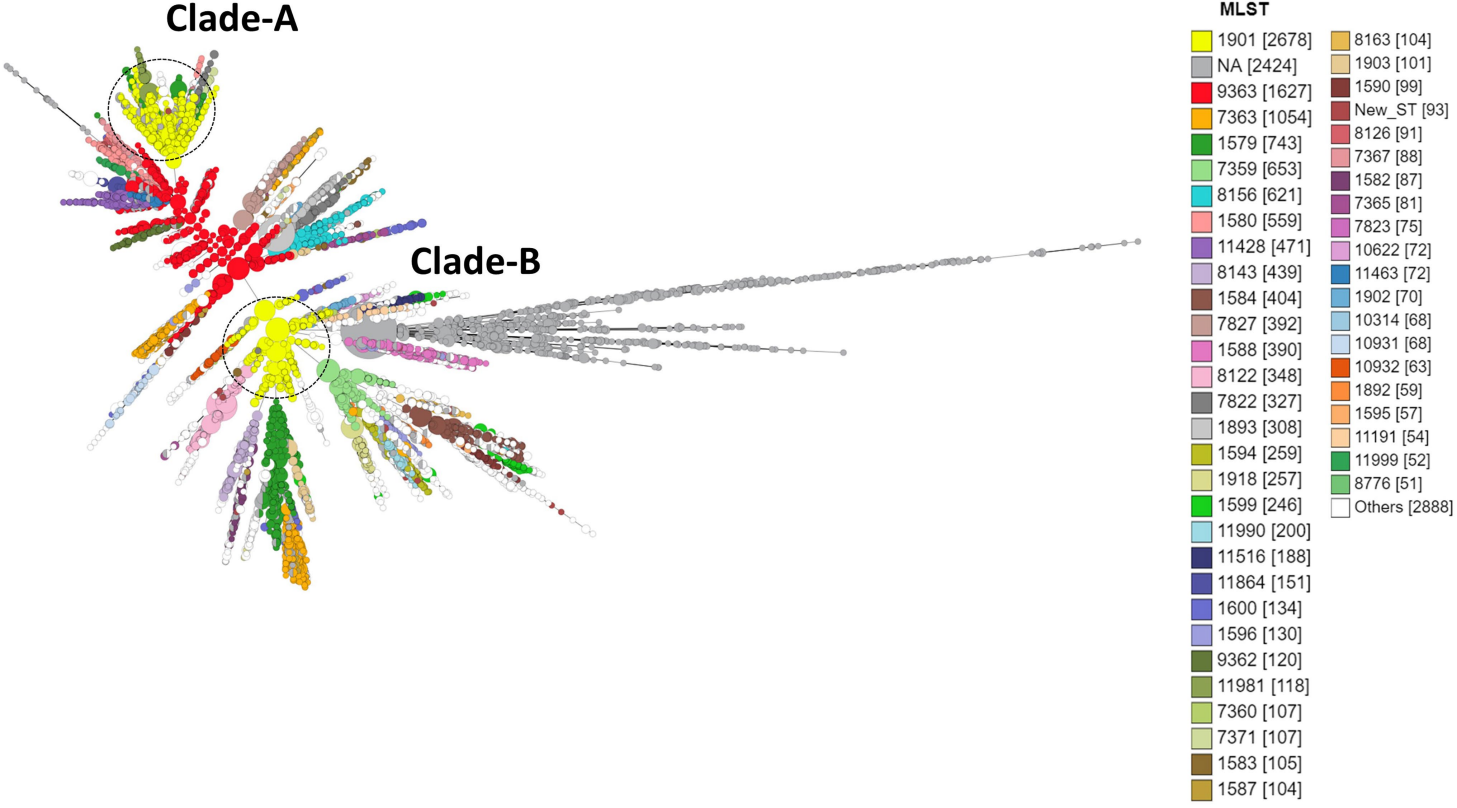
Minimum spanning tree comparing core-genome allelic profiles with MLST resulting in isolates with similar allelic profiles forming clusters. Isolates are displayed as circles. The size of each circle indicates the number of isolates of this particular type. Numbers in brackets refer to the number of isolates. Clade A, B of ST1901 are represented as dotted circles.

A total of 1,323 loci were defined as core to the *Neisseria* genome in this dataset. Allelic designations were defined for approximately 95% of the core-genome. A total of 583 different sequence types (STs) characterized the available population. The most frequent ST was ST1901 (*n* = 2,677) followed by ST9363 (*n* = 1,627), ST7363 (*n* = 1,054), ST1579 (*n* = 743), and ST7359 (*n* = 653; Figure 6; Supplementary Table 2 in Table 2). MLST types were not available for 2,424 isolates, and 93 isolates were classified as new ST types. Of the 2,384 CIP HLR isolates detected, half of the isolates [1,201 (50.4%)] belonged to ST1901, followed by ST7363 (11.49%) and ST1579 (2.5%). ST types were not available for 231 (9.6%) isolates. The total number of HLR, I, R, and S isolates and their prevalence in each continent is listed in Supplementary Table 4 in Data Sheet 1.

The three most prevalent ST types (>1,000 isolates) were further characterized. ST1901 was split into two clades, referred to as Clade A and Clade B (Figure 6). Isolates of clade A were predominately from North America, while clade B isolates were predominately from Europe (Figure 2). Both clades had substitutions at GyrA-S91F (*n* = 2,616, Figure 4A; Supplementary Table 2 in Table 2), GyrA-D95G (*n* = 2,585, Figure 4C), and ParC-S87R (*n* = 2,663, Figure 5C). Around 0.4, 20.5, and 44% of isolates belonging to ST1901 were S, R, and HLR, respectively. About 34% of isolates had no MIC information (Table 1).

**Table 1:** Distribution of 5 predominant ST-types of N. gonorrhoeae isolates, country of isolation and their known resistance-associated mutations.

0	CIP resistance	Africa	Asia	Europe	North America	Oceania	South America	NA	Total no. of isolates
1579			56	251	197	219	1	19	743
	HLR		51	4	4			2	61
	I			1	2				3
	R		4	6	21	5		1	37
	S			91	78	214	1	16	400
	NA		1	149	92				242
1901			152	1073	1007	126		284	2677
	HLR		75	322	567	31	24	182	1201
	R		41	102	235	86	11	76	551
	S			11	1			1	13
	NA		36	638	204	9		25	912
7359			20	150		483			653
	R			1					1
	S		20	126		296			442
	NA			23		187			210
7363		1	237	446	85	252	1	28	1050
	HLR		82	164	15	2	1	10	274
	R		47	88	12	230		9	386
	S	1	2	12		13			28
	NA		106	182	58	7		9	362
9363				597	548	418	9	55	1627
	HLR			16	11			1	28
	I			7	4				11
	R			6	29	4		1	40
	S			287	335	412	9	52	1095
	NA			281	169	2		1	453

ST9363 isolates were distributed across Europe (36.69%), North America (33.68%), and Oceania (25.69%; Table 1; Figure 2). The first HLR isolate was found in North America in 2011. About 67.3, 0.6, 2.4, and 1.7% of isolates were S, I, R, and HLR, respectively. About 27.8% of isolates had no MIC information. The resistant isolates had one or more of the following substitutions: GyrA-S91F (*n* = 78, Figure 4A), GyrA-D95A/G (*n* = 76/2, Figure 4C), ParC-D86N (*n* = 71, Figure 5B), and the ParC-S87R (*n* = 10, Figure 5C).

At least three clades for ST7363 were observed (Figure 6). Isolates that belonged to ST7359 were mostly from Oceania (Figure 2). Out of the 653 isolates, 442 were susceptible, and MIC was not available for 210 isolates. Only one isolate from Europe (Ireland) was CIP resistant (MIC-2 mg/L) with no known RAMs. Further details pertaining to the distribution of MLST, CIP MICs, and the prevalence in each continent are provided in Table 1 and Supplementary Table 4 in Data Sheet 1.

### The Time of Emergence of Fluoroquinolone Resistance-Associated Mutations in *Neisseria gonorrhoeae*

**Figure 7:**
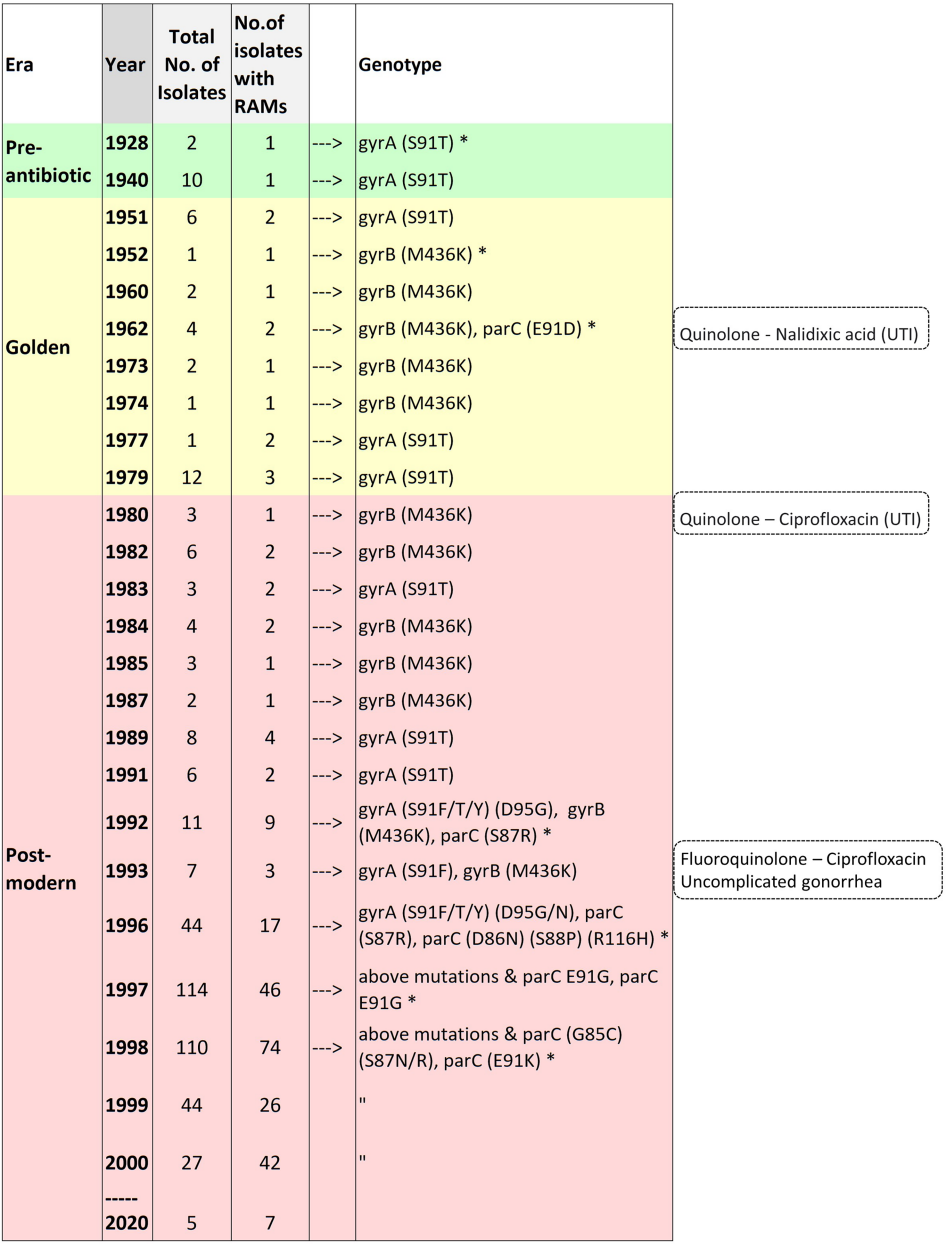
Time of emergence of ciprofloxacin resistance-associated mutations. Asterisks denote the first occurrence of the mutation.

Substitution at position 91 (S91T) of GyrA was first observed in one of two of the oldest isolates in the collection obtained in 1928. The MICs were not available for the pre-antibiotic and the golden era isolates. GyrA S91F, which leads to high-level resistance to CIP, was first observed in 1992 (post-modern era). Four isolates were available from the same year. Two isolates were from the Philippines and had GyrA-S91F along with additional substitutions, and two isolates had no RAMs. Out of the two isolates with RAMs, one isolate belonged to ST1901 (D95G; ParC-S87R), and the other to ST7367 (D95G). Substitutions at position 95 of GyrA (D95G/N) and positions 86, 87, 88, and 91 of ParC were first observed in 1996. A substitution in position 85 of ParC was first observed in 1998 (Figure 7; Supplementary Table 6 in Data Sheet 1).

### Prevalence of HGT in QRDR Region of *gyrA*, *gyrB*, *parC*, and *parE*

The lengths of *gyrA*, *gyrB*, *parC*, and *parE* of the reference isolate FA1090 were 2,751, 2,391, 2,307, and 1,986 bp, respectively. The length of the alleles ranged between 2,454–3,054 bp for *gyrA*, 2,388–2,644 bp for *gyrB*, 2088–2,538 bp for *parC*, and 1,629–2,151 bp for *parC*. Multiple alignment files of each gene were trimmed to the gene length of the FA1090 isolate and were used in the recombination analysis.

**Figure 8:**
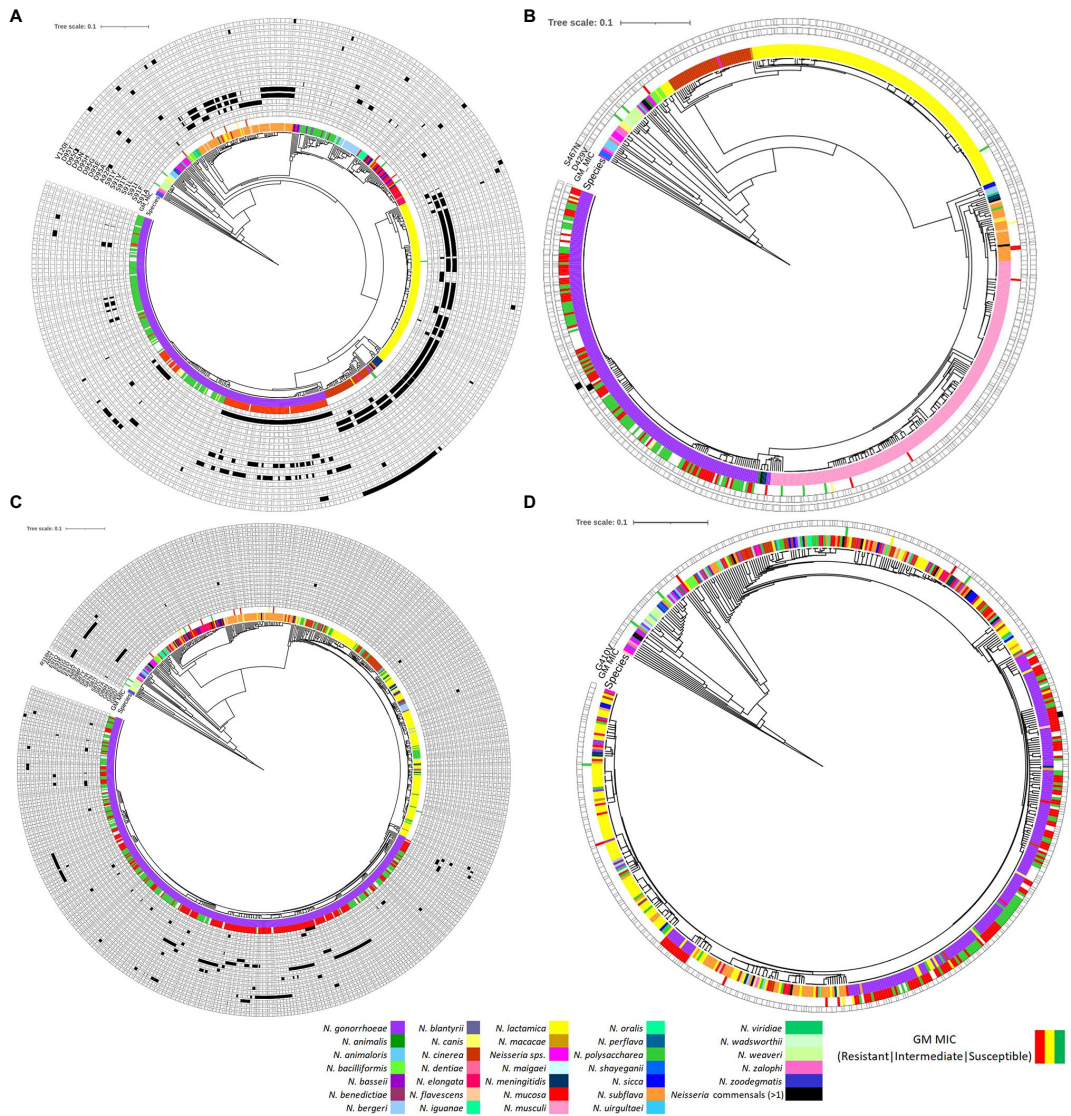
Phylogenetic trees of **(A)** *gyrA*, **(B)** *gyrB*, **(C)** *parC*, and **(D)** *parE* based on *Neisseria* spp. Figure was generated by iTOL using cgMLST profile data determined by the chewBACCA software. Purple node denotes *N. gonorrhoeae*, and the rest denotes *Neisseria* commensals. Filled and outlined black rectangle denotes the presence and absence of non-synonymous SNPs, respectively. The geometric mean (GM) ciprofloxacin MIC SIR profiles are denoted as green, yellow, and red, respectively.

**Figure 9:**
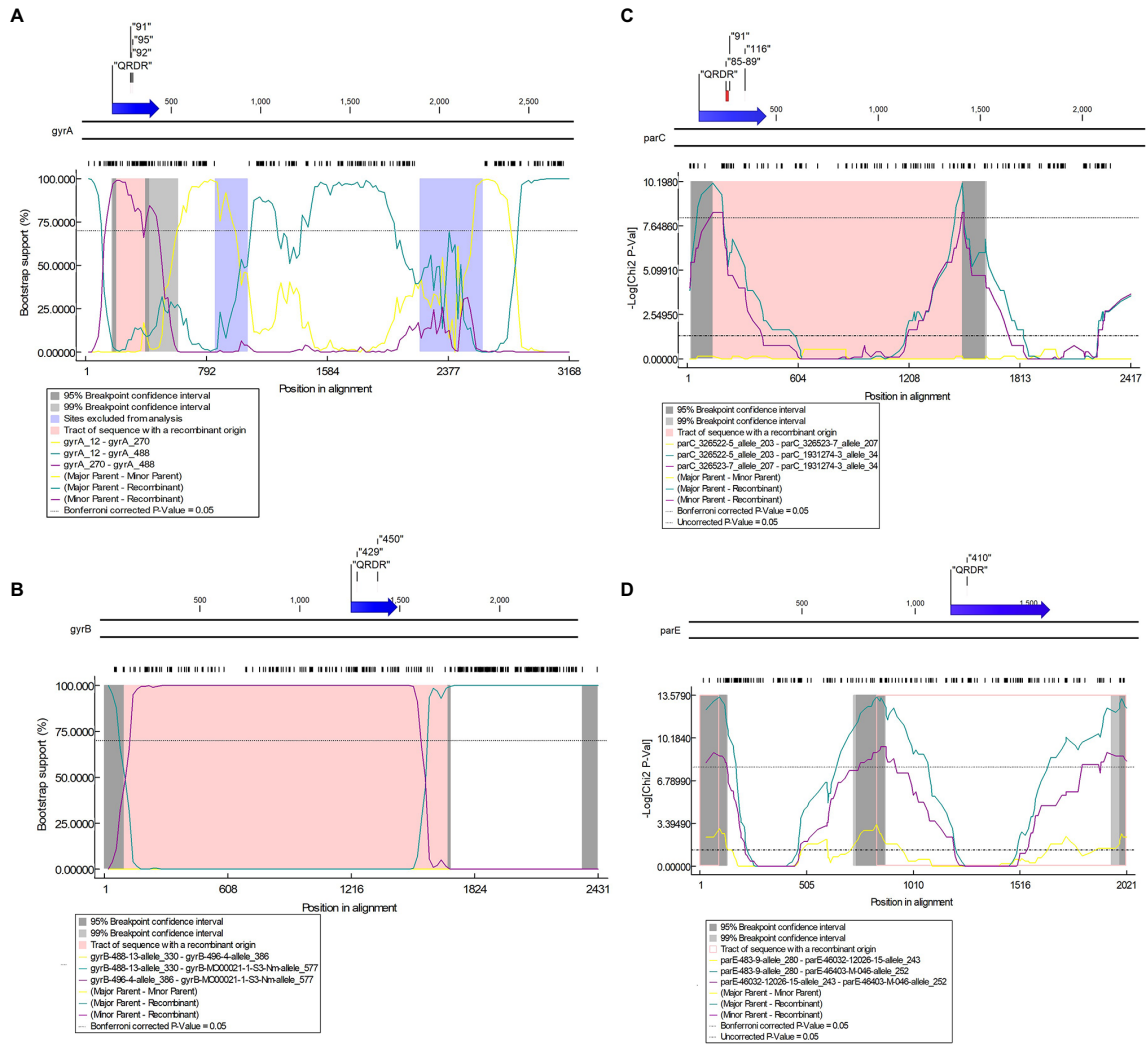
Potential putative recombination events between *Neisseria* strains. **(A)** Recombination event 3 in *gyrA* between *N. gonorrhoeae* (recipient) and *N. lactamica* (donor); **(B)** Recombination event 3 in *gyrB* between *N. gonorrhoeae*/*N. meningitidis* (recipient) and *N. macacae* (donor); **(C)** Recombination event 21 in *parC* between *Neisseria* sp. (recipient) and *N. zoodegmatis* (donor); **(D)** Recombination event 19 in *parE* between *N. gonorrhoeae* (recipient) and *N. mucosa* (donor). Top—The functional map of *N. gonorrhoeae* reference strain FA1090 with QRDR and known resistance-associated mutations (RAMs) are shown within inverted commas. Bottom—Bootscan **(A,B)** and maxchi **(C,D)** plots generated using RDP4 software are depicted.

About 688, 631, 898, and 595 alleles were found for *gyrA*, *gyrB*, *parC*, and *parE* genes, respectively (Figures 8A–D; Supplementary Tables 6A–D in Data Sheet 1). Putative HGT events were predicted in all the genes (Figures 9A–D). Unique recombination events were supported by at least two out of seven detection methods. A total of 63, 8, 80, and 67 recombination events were identified for *gyrA*, *gyrB*, *parC*, and *parE*, respectively. For the sequences that included only the RAMs in *gyrA* and *parC*, a total of 58 recombination events were identified for *gyrA* (Supplementary Table 7E in Data Sheet 1) and 16 for *parC* gene (Supplementary Table 7F in Data Sheet 1). Each recombination event was checked manually. Examples of recombinant events with known RAMs in QRDR regions of *gyrA* and *parC* and in QRDR regions of *gyrB* and *parE* are presented below.

#### gyrA

For *gyrA*, the QRDR region is present between 169 and 432 bp. Putative recombination event 32 in the QRDR region with *N. gonorrhoeae* as a recombinant was identified. The event was supported by five methods (RDP, GENECONV, Bootscan, Maxchi, and Chimaera). *Neisseria gonorrhoeae* (allele—488; *n* = 2, GM MIC-0.02 mg/L, ST11702, Year-1940) was the recombinant, and evidence of the same recombinant event was present in four other alleles (allele-93, *n* = 3, ST1175, Year-2010–2012; allele-491; *n* = 2, ST1688, Year-1928; allele-496, *n* = 2, ST11703, Year-1951; and allele-508, *n* = 2, ST11708, Year-1951). The corresponding minor parent was *N. lactamica* (allele-270; *n* = 1; recombinant region—197–396 nt), and major parent was *N. cinerea* (allele-12; *n* = 1; recombinant region—1–199 and 397–3,168 nt; Figure 9A; Supplementary Table 7A in Data Sheet 1; Supplementary Figure 2). The minor parent *N. lactamica* and the recombinants had the S91T substitution (Supplementary Table 6A in Data Sheet 1).

Recombination events were also found in the commensal *Neisseria*. In event 5, for example, *N. polysaccharea* (allele—361; *n* = 1) was the recombinant, and the sequences with the same recombinant event were present in 10 isolates belonging to five alleles; *N. bergeri* (alleles- 28, 214, 215, and 223; *n* = 9) and *N. oralis* (allele-29, *n* = 1). The corresponding minor parent was *N. cinerea* (allele-61; *n* = 1; recombinant region—1–172 and 2,946–3,168 nt) and major parent was *N. elongata* (allele-637; *n* = 2; GM MIC-0.25 mg/L) recombinant region—173–2,945 nt (Supplementary Table 7A in Data Sheet 1). The major parent *N. elongata* (allele-637), the recombinant *N. polysaccharea* (allele—361), and isolates with the same recombinant (*n* = 10) had the S91V substitution (Supplementary Table 6A in Data Sheet 1). The event was supported by all seven methods.

#### gyrB

The QRDR region of *gyrB* is between 1,255 and 1,488 bp, which is also the zoliflodacin resistance-conferring region (Figure 9B). Recombination event 3 was supported by all seven methods (RDP, GENECONV, Bootscan, Maxchi, Chimera, SiSscan, and 3Seq; Figure 9B) and *N. meningitidis* (*n* = 1; allele-577) was the recombinant. The same recombination event was present in five alleles, including *N. gonorrhoeae* (*n* = 13, allele-472, 518, and 562) and *N. meningitidis* (*n* = 2; allele-544 and 567). The minor and major parents were *N. macacae* (allele-386; *n* = 1; region—96–1,690 nt) and *N. mucosa* (allele-330; *n* = 1; region—1–95 and 1,691–2,431 nt), respectively (Figure 9B; Supplementary Tables 5, 7B in Data Sheet 1). The recipient and the donor had no known RAMs (Supplementary Table 6B in Data Sheet 1).

#### parC

The QRDR region lies between 121 and 452 bp. Recombination event 21 took place in the QRDR region and involved the transmission of known RAMs between commensal *Neisseria*. The recombinant was *Neisseria* sp. (allele—34; *n* = 2). *Neisseria animaloris* (allele—203; *n* = 1; recombinant region—1–141 and 1,499–2,417 nt) and *N. zoodegmatis*. (allele—207; *n* = 1; recombinant region—142–1,498 nt) were the major and minor parents, respectively (Figure 9C). The same event was seen in another allele (allele-35, *n* = 2). The minor parent and the recombinant had the S87A and E91A RAMs (Supplementary Table 6C in Data Sheet 1). The event was supported by three methods (Maxchi, Chimera, and 3Seq).

#### parE

The QRDR region lies between ~1,140 and 1,600 bp. Recombinant event 19 for *parE* was supported by three methods (Maxchi, Chimera, and 3Seq). The recombinant was *N. gonorrhoeae* (allele-252; *n* = 1). The corresponding major parent was *N. cinerea* (allele 280, *n* = 1; recombinant region—92–837 nt), and the unknown parent was inferred from *N. mucosa* (allele-243; *n* = 3; recombinant region—1–91 and 838–2,021 nt; Figure 9D). The recipient and the donor had no known RAMs (Supplementary Table 6D in Data Sheet 1).

## Discussion

The large and diverse collection of *Neisseria* isolates used in this study enabled us to investigate the temporal and spatial patterning of fluoroquinolone RAMs as well as the evidence of HGT in *gyrA*, *gyrB*, *parC*, and *parE* genes in *N. gonorrhoeae* and related *Neisseria* spp.

In accordance with previous studies, we found that the global spread of ciprofloxacin-resistant *N. gonorrhoeae* is associated with three STs 1,901, 9,363, and 7,363 being responsible for 65% of all high-level resistance to ciprofloxacin (Osnes et al., 2020; Reimche et al., 2021). ST1901, with substitutions in GyrA-S91F, D95G, and ParC-S87R, was by itself responsible for over half of all high-level resistance. Osnes et al. have recently demonstrated that this ST emerged in East Asia before spreading globally between 1987 and 1996 (Osnes et al., 2021). Its emergence and spread were facilitated by the increasing use of fluoroquinolones as a treatment for gonorrhea at this time (Osnes et al., 2020). The widespread use of fluoroquinolones in the general population may also have played a role (Kenyon et al., 2018a). Notably, no global distribution of ST1901 clones with substitutions in GyrA-S91Y and ParC-R116H during the same period from East Asia was observed.

The GyrA-S91T substitution was first reported in 1928 in *N. gonorrhoeae* and cannot be plausibly linked with antimicrobial pressure. GyrA-S91T is not associated with reduced susceptibility to ciprofloxacin (Golparian et al., 2020). Notably, two isolates from 1928 had different amino acid substitutions at position 91. One isolate belonging to ST11688 had the threonine (T) amino acid at position 91, whereas the other isolate from ST11680 had the serine (S) at position 91. Interestingly, the meningococci have the threonine (T) substitution at position 91 (Hong et al., 2013). Resolving the parental isolate of gonococci could resolve the ST types of gonococci, especially the two clades of ST1901. Further analysis pertaining to the above was not carried out as this was beyond the scope of the present study. The next mutation to occur in the four genes under consideration was a substitution in ParC-E91D in 1962 in *N. canis*. This occurred at the time when the first-generation quinolone, nalidixic acid, was introduced (Lesher et al., 1962).

Our analyses suggest that 199 bp of the QRDR region in *gyrA* with GyrA-S91T substitution was acquired from *N. lactamica*. Fourteen isolates of *N. gonorrhoeae* belonging to five alleles had obtained this section of the QRDR region, including the GyrA-S91T. All the isolates belonged to the pre-antibiotic/golden era except three isolates from allele-93 that belonged to the post-modern era with a GM MIC of 0.006 mg/L. Evidence of HGT in QRDR regions of *Neisseria* spp. and mutation harboring commensals with substitutions in known amino acid positions, GyrA-S91, ParC-S87, and ParC-E91 were identified. The recombinants with GyrA-S91V and ParC-S87A, ParC-E91A were *N. polysaccharea* and *Neisseria* sp., respectively. The mutation harboring commensal of GyrA-S91V in the recombinant *N. polysaccharea* (*n* = 1, MIC-NA) was *N. elongata* (*n* = 2, GM MIC—0.25 mg/L) and ParC-S87A, E91A in the recombinant *Neisseria* sp. (*n* = 2, MIC-NA) was *N. zoodegmatis* (*n* = 1, MIC-NA). Twenty-one *Neisseria* commensal isolates had the GyrA-S91V substitution, but only one isolate, *N. elongata* had the data for ciprofloxacin susceptibility (MIC 0.25 mg/L). It was therefore not possible to determine if the mutation is associated with reduced susceptibility to ciprofloxacin. The same implies to the substitutions in ParC.

The analysis of whole-genome sequences identified non-synonymous mutations in the GyrB-D429V and S467N, that is, where zoliflodacin first- or second-step resistance mutations have been previously selected *in vitro* (Alm et al., 2015; Foerster et al., 2015, 2019). Substitutions in GyrB-D429V was present in one isolate belonging to ST7363 (Adamson et al., 2021; Unemo et al., 2021). GyrB-S467N substitution was present in 50 isolates and belonged to ST7367 (*n* = 1), ST7363 (*n* = 37), and ST1925 (*n* = 12). Evidence of HGT in the QRDR region of *gyrB* was identified, and the commensal donor was *N. macacae*.

Caveats of our study include that only a few early isolates were available, and very few isolates from some continents, such as Africa and South America, were available. The study included only nine *N. meningitidis* isolates. The probability of detecting HGT events could have been maximized using a greater number of *N. meningitidis*. Moreover, MIC values were not available for many commensal isolates. Some of the Pathogenwatch MICs are estimated based on genotype using 485.toml.^5^ These estimated MICs may differ slightly from MICs obtained *via* phenotypic testing. We were also not able to identify the commensal donors for *parC* RAMs. This could be due to the paucity of commensal isolates with known RAMs. We did not include other genetic mutations, such as the *norM* promoter mutation, which can lead to increased fluoroquinolone resistance (Rouquette-Loughlin et al., 2003). Finally, the HGT events were not confirmed experimentally.

Studies have shown that chromosomal *gyrA* and *parC* mutations from commensal *Neisseria* have been responsible for most fluoroquinolone resistance in meningococci (Chen et al., 2020; Shen and Chen, 2020). With the current dataset, we showed evidence of HGT in genes *gyrA*/*B* and *parC*/*E* and found that HGT played a lesser role in acquiring fluoroquinolone resistance mutations in gonococci. Nevertheless, our findings add to the growing evidence base that commensal *Neisseria* species act as a reservoir of antimicrobial resistance that can be taken up by the pathogenic *Neisseria* (Bowler et al., 1994; Shafer, 2018; Yahara et al., 2018; Fiore et al., 2020; Goytia et al., 2021; Manoharan-Basil et al., 2021). The study highlights the significant role that HGT can play in transferring QRDR regions to *N. gonorrhoeae*. Zoliflodacin is a promising new drug to treat *N. gonorrhoeae* (Foerster et al., 2019; Bradford et al., 2020; Adamson et al., 2021; Kenyon et al., 2021). Our finding that zoliflodacin resistance-conferring region of *gyrB* in *N. gonorrhoeae* can be taken up from *N. macacae* has important consequences. Extensive use of zoliflodacin could lead to resistance in commensal *Neisseria*, which could then be transferred to *N. gonorrhoeae via* transformation. This provides an additional rationale to conduct surveillance of antimicrobial susceptibility in *Neisseria* commensals to prevent the emergence of AMR in the pathogenic Neisseria (Kenyon et al., 2018b; Dong et al., 2020; Fiore et al., 2020).

## Data Availability Statement

The original contributions presented in the study are included in the article/Supplementary Material, further inquiries can be directed to the corresponding author.

## Author Contributions

SM-B and CK conceptualized the study, interpreted the data, performed the statistical analysis, and wrote the first draft. SM-B was responsible for data collection and bioinformatic analysis. All authors read the final draft and approved the submitted version.

## Funding

The study was funded by SOFI 2021 grant—“PReventing the Emergence of untreatable STIs *via* radical Prevention” (PRESTIP).

## Conflict of Interest

The authors declare that the research was conducted in the absence of any commercial or financial relationships that could be construed as a potential conflict of interest.

## Publisher’s Note

All claims expressed in this article are solely those of the authors and do not necessarily represent those of their affiliated organizations, or those of the publisher, the editors and the reviewers. Any product that may be evaluated in this article, or claim that may be made by its manufacturer, is not guaranteed or endorsed by the publisher.

## Footnotes

^1^ https://www.ncbi.nlm.nih.gov/genbank/

^2^ https://pathogen.watch/ngonorrhoeae

^3^ http://www.eucast.org/clinical_breakpoints/

^4^ https://www.uniprot.org/

^5^ https://gitlab.com/cgps/pathogenwatch/amr-libraries/blob/0.0.17/485.toml

## Supplementary Material

Supplementary Presentation 2 Alignment of *GyrA* alleles-1 (reference, FA1090), 3, 496, 488, 491, 508 from *N. gonorrhoeae* and allele-270 from *N. lactamica* (donor). The quinolone resistance determining region (QRDR), the 199 bp of the recombinant region and the resistance associated mutations (RAM) are depicted in red, green and blue arrows, respectively. Amino acid substitutions are indicated as grey shading.
