# Supplementary material for: Horizontal Gene Transfer of Fluoroquinolone Resistance-Conferring Genes From Commensal Neisseria to Neisseria gonorrhoeae: A Global Phylogenetic Analysis of 20,047 Isolates
Source: Front Microbiol. 2022 Mar 17;13:793612. doi: 10.3389/fmicb.2022.793612 (PMC8973304; doi:10.3389/fmicb.2022.793612)
Supplement: Supplementary file 2 [file Presentation_1.pptx]

## Slide 1
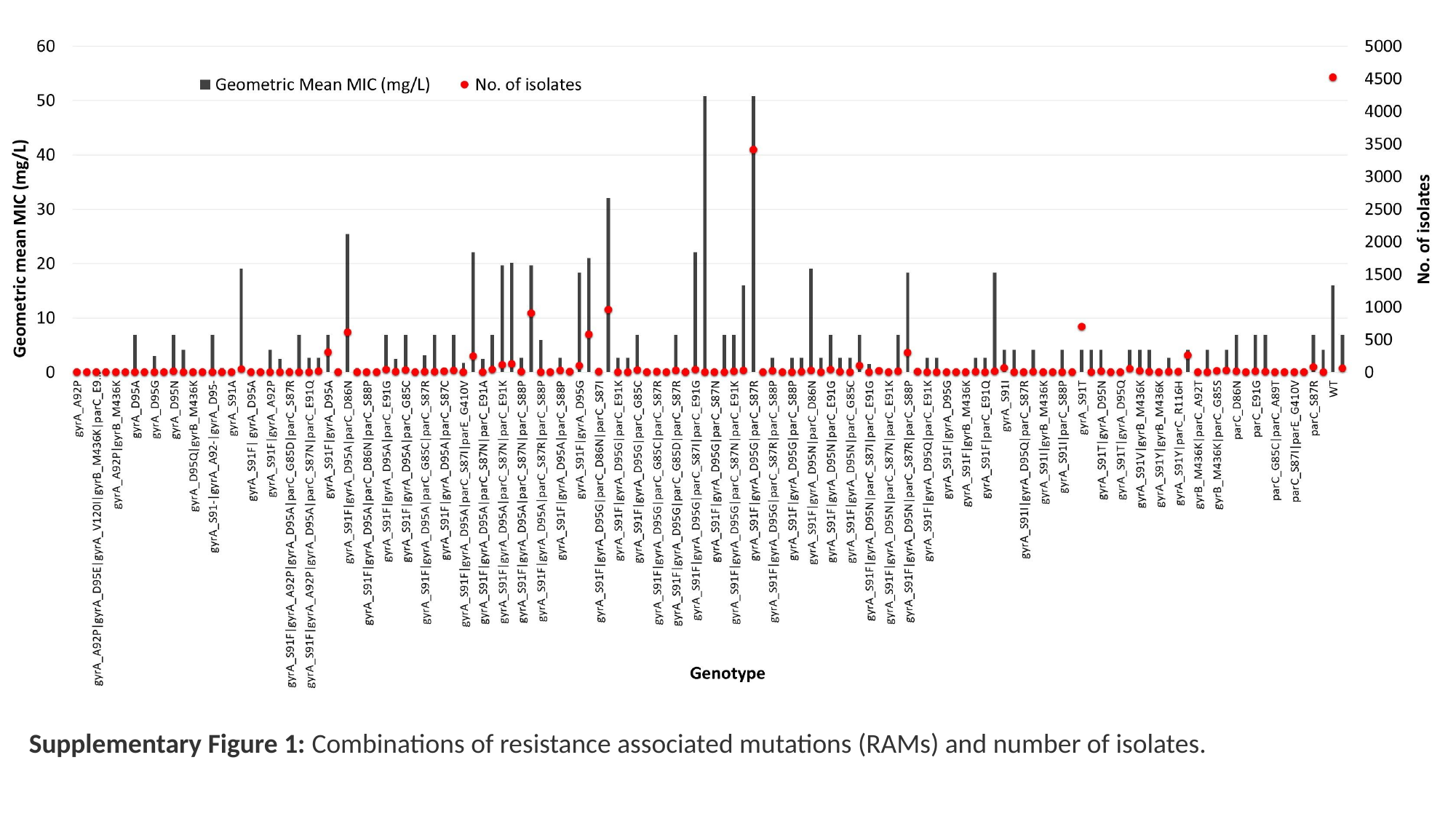

Supplementary Figure 1: Combinations of resistance associated mutations (RAMs) and number of isolates.
