# Supplementary figures and images for: Horizontal Gene Transfer of Fluoroquinolone Resistance-Conferring Genes From Commensal Neisseria to Neisseria gonorrhoeae: A Global Phylogenetic Analysis of 20,047 Isolates
Source: Front Microbiol. 2022 Mar 17;13:793612. doi: 10.3389/fmicb.2022.793612 (PMC8973304; doi:10.3389/fmicb.2022.793612)

1

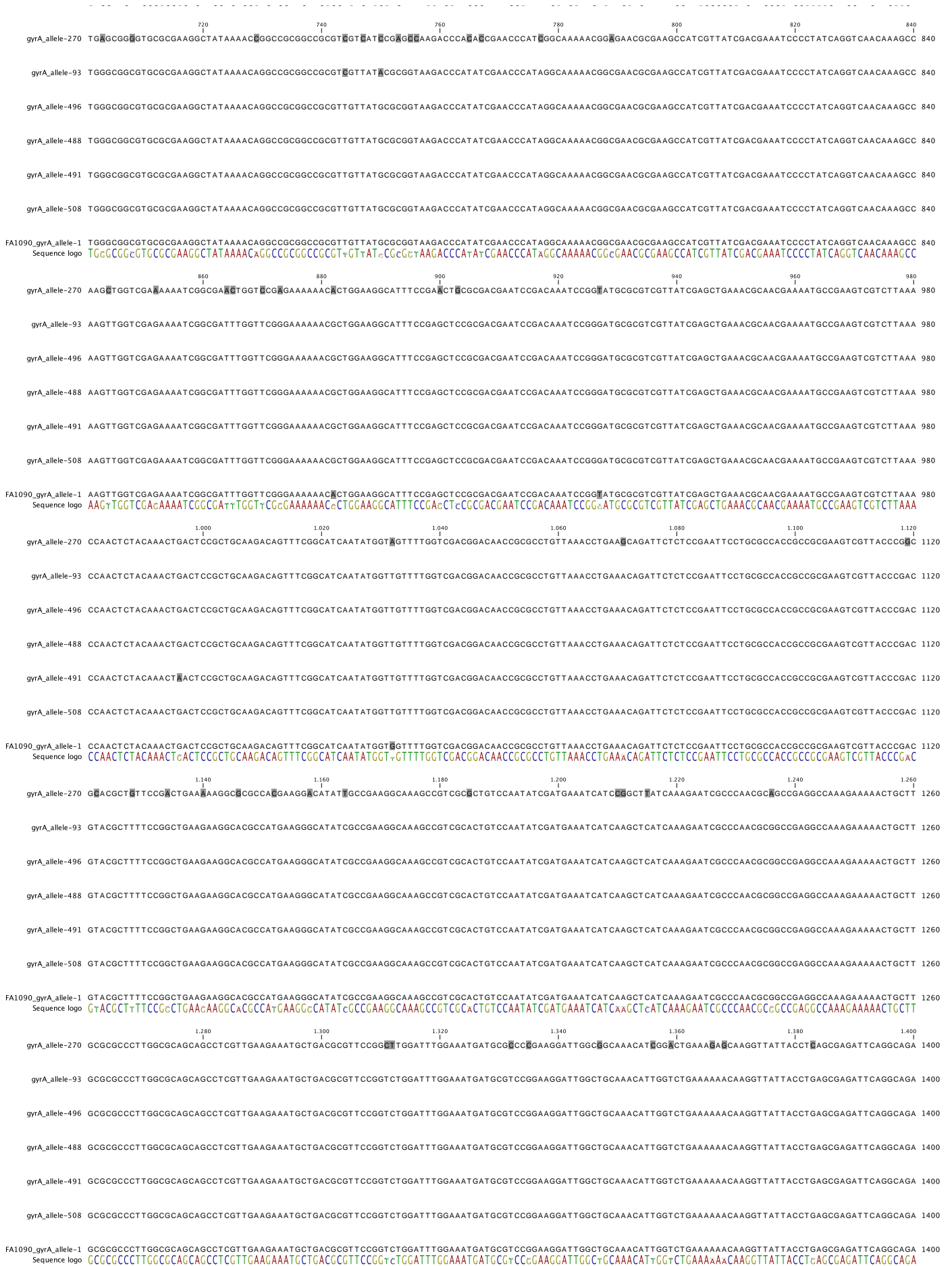

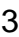

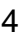

Supplement: Supplementary Presentation 2 — Alignment of GyrA alleles-1 (reference, FA1090), 3, 496, 488, 491, 508 from N. gonorrhoeae and allele-270 from N. lactamica (donor). The quinolone resistance determining region (QRDR), the 199 bp of the recombinant region and the resistance associated mutations (RAM) are depicted in red, green and blue arrows, respectively. Amino acid substitutions are indicated as grey shading. [file Presentation_2.pdf]
